# Lung cancer in the Kashmir valley

**DOI:** 10.4103/0970-2113.68309

**Published:** 2010

**Authors:** Parvaiz A. Koul, Satish Kumar Kaul, Mohammad Mushtaq Sheikh, Reyaz A. Tasleem, Azra Shah

**Affiliations:** *Department of Internal and Pulmonary Medicine, SKIMS, Srinagar, Kashmir, India*; 1 *Department of Pathology, Government Medical College, Srinagar, Kashmir, India*; 2 *Department of Pathology, SKIMS, Srinagar, Kashmir, India*

**Keywords:** Cancer, epidemiology, India, lung

## Abstract

**Background:**

Lung cancer has been found to be the second commonest cancer according to a hospital-based data from Kashmir, India. However, no incidence studies are available.

**Objective:**

To ascertain the incidence of lung cancer in Kashmir.

**Materials and Methods:**

All newly histologically diagnosed cases of lung cancer seen in various hospital and private laboratories of the Kashmir valley were registered over a period of two years (January 1, 2004 to December 31, 2005). Also included were patients attending the various oncological service areas of the institute and those diagnosed from any other laboratory outside the state. The incidence rate was calculated using the January 2005 population as the reference population estimated using the census-based projected populations.

**Results:**

Four hundred and sixty–two incident cases of lung cancer were seen during the study period. The crude incidence rate, age standardized (world) and truncated age adjusted (40-69 years, world) incidence rates for lung cancer per 100 000 population were 4.01, 6.48 and 15.28 respectively (males 6.55, 10.09 and 23.94 respectively and females 1.19, 2.14 and 4.65). The age adjusted rates for males in district Srinagar was 19.34 per 100 000. One hundred and fifty nine (69.8%) of the 221 had a history of Hukkah smoking.

**Conclusions:**

Even though Kashmir as a whole is a low incidence area for lung cancer (ASR of < 15), Srinagar district has the highest incidence of lung cancer among the males in Kashmir. The data presented is assumed to be the closest approximation to a population-based data registry and the geographical incidence maps of ICMR need appropriate updating

## INTRODUCTION

Lung cancer is one of the most common malignant neoplasms worldwide, accounting for more deaths than any other cancer cause. Although it was considered to be uncommon at the beginning of the century, it has reached epidemic proportions and is currently the leading cause of cancer-related deaths in the Western countries. It was initially thought to be extremely infrequent in India. Lung cancer constituted 14.4% of all cancers according to a review of 9210 consecutive autopsies reported by Banker in 1957.[1] Viswanathan collected information from different hospitals of the country and found that the incidence of lung cancer in hospital population was 27.4 per million in 1950 and 78.6 per million in 1959. He also found an increase in the incidence of the bronchogenic carcinoma over a period of 10 years from 16.1 in 1950 to 26.9 in 1959 per 1000 malignancies.[2]

The National Cancer Registry Programme of ICMR, which collected data from six different parts of the country, reported that cancer of the trachea, bronchus and lungs was the most common type of malignancy in males in 1989 from Bombay, Delhi and Bhopal. It was the second most common in Madras and third in Bangalore and was most unusual in Barshi, a rural area.[3] As per the data given in first All India Report (AIR) 2001-2002; in males the lung cancer is the leading site of cancer in 8 of the 12 population-based cancer registries (PBCRS), namely Bhopal, Delhi, Mumbai, Ahmedabad, Kavengapally and Calcutta.[4] Lung cancer was reported to be the second most common malignancy in an earlier hospital-based study from Kashmir Valley of the Indian subcontinent.[5] Hukkah smoking was found to be highly prevalent in the lung cancer patients of another small study of 25 hospitalized patients.[6] The population in Kashmir is largely Muslim and the present study is the first multi institutional study on the incidence of lung cancer in Kashmir.

## MATERIALS AND METHODS

All newly diagnosed and histologically proven cases of lung cancer over a period of two years (Jan 2004- Dec 2005) were registered. The registration process included the following:

- Assessment of records of histopathological labs of Sher-i-Kashmir Institute of Medical Sciences and Govt. Medical College, Srinagar. The Sher-i-Kashmir Institute of Medical Sciences is a 650-bedded tertiary hospital cum referral center which constitutes the main oncology referral center in the valley of Kashmir. A small number of patients diagnosed at other hospitals who may not seek treatment keep attending these hospitals and their records were obtained from these hospitals. Most of the patients are hence diagnosed in the institute and the histopathology is available from the histopathology department. SMHS hospital is the other major hospital in the valley and patients are diagnosed in this hospital also, but the patients are generally referred to the SKIMS for treatment. However, the data of the patients who continue to follow this hospital were also recorded.
- Assessment of the records of different private pathological laboratories in the valley where very few samples are processed or got processed from the other laboratories of the country. All laboratories that have the facilities for histopathology (all located in Srinagar) were contacted and their data were obtained on a fortnightly basis. Patients whose biopsies were reported from outside the state from labs like Lal Path Labs, Ranbaxy Labs and Micro were included after a regular inquiry from these labs. No lab was left out.
- Assessment of the records of various departments like Internal Medicine, Cardiovascular and Thoracic surgery, Radiation oncology and Medical Oncology of SKIMS, Srinagar which could be the only points of contact of patients diagnosed with lung cancer.
  The assessment was made on a fortnightly basis over the study period. The information about the cancer patients was collected and recorded on a predefined proforma. Various definitions, statistical terms and methods used in calculation for the present study are the following:

1. **Lung cancer cases**
  Patients having malignant neoplastic growth of trachea, bronchus and lungs as defined by the International classification of Disease - Oncology (3^rd^ ed, WHO 2000) ICD33 and ICD34 respectively and considered reportable and therefore registered.
2. **Age group**
  The age groups used for estimating population as well as grouping cancer cases were 0-9, 10-19, 20-29,..and 80+
3. **Incidence**
  It denotes new cancer diagnosed in a defined population in a specified time period. For this study all lung cancer cases diagnosed histologically from 1st Jan 2004 to 31st Dec 2005 in Kashmir valley are included.
4. **Rates**
  Rates of cancers have been expressed per 100 000 population.
5. **Crude incidence rate (CR)**
  This refers to the rate obtained by the division of total number of cancer cases by the corresponding estimated population (mid-year) for that respective geographic area and multiplication of the obtained value with 100 000.
  $$\mathrm{CR}\;=\;\frac{\mathrm{New}\;\mathrm{cases}\;\mathrm{of}\;\mathrm{cancer}\;\mathrm{of}\;a\;\mathrm{particular}\;\mathrm{year}}{\mathrm{Estimated}\;\mathrm{population}\;\mathrm{of}\;\mathrm{the}\;\mathrm{same}\;\mathrm{year}}\;\times \;100\;000$$
6. **Age specific rate (ASPR)**
  This refers to the rate obtained by the division of the total number of cancer cases by the corresponding estimated population in that age group and sex/site/geographic area/time period and multiplication of the obtained value with 100 000.
  $$\mathrm{ASPR}\;=\;\frac{\mathrm{New}\;\mathrm{cases}\;\mathrm{of}\;\mathrm{cancer}\;\mathrm{of}\;a\;\mathrm{particular}\;\mathrm{year}\;\mathrm{in}\;\mathrm{the}\;\mathrm{given}\;\mathrm{age}\;\mathrm{group}}{\mathrm{Estimated}\;\mathrm{population}\;\mathrm{of}\;\mathrm{the}\;\mathrm{same}\;\mathrm{year}\;\mathrm{for}\;\mathrm{the}\;\mathrm{given}\;\mathrm{age}\;\mathrm{group}}\;\times \;100\;000$$
7. **Age adjusted or age standardized rate (ASR)**
  In our study world standard population approximates the proportional age distribution of the world. In the present study ASR is calculated according to a direct method (Boyle and Parkin 1991) by obtaining the age specific rates and applying these to the standard population in that age group.
  $$\mathrm{ASR}\;=\;\frac{\Sigma \;(\mathrm{ASPR})\;\times \;(\mathrm{No}.\;\mathrm{of}\;\mathrm{persons}\;\mathrm{in}\;\mathrm{standard}\;\mathrm{world}\;\mathrm{population}\;\mathrm{in}\;\mathrm{that}\;\mathrm{age}\;\mathrm{group})}{100\;000}\;$$
8. **Census and population estimation**
  The ten year age group population of 1991 and total population of Kashmir valley in six districts as per 2001 census have been used in this study to calculate the estimated population of January 2005 as per arithmetic progression method.
  January 2005 population = Census 1991 + 10 year increase in population + 48 months increase in population
  $$10-\mathrm{year}\;\mathrm{increase}\;\mathrm{in}\;\mathrm{population}\;=\;\mathrm{Relative}\;\mathrm{proportion}\;\mathrm{of}\;\mathrm{population}\;1991\;\mathrm{for}\;\mathrm{each}\;\mathrm{specific}\;\mathrm{age}\;\mathrm{group}\;\times \frac{\mathrm{Census}\;2001-\mathrm{Census}\;1991}{100}$$
  $$48\;\mathrm{months}\;\mathrm{increase}\;\mathrm{in}\;\mathrm{population}\;=\;10-\mathrm{year}\;\mathrm{increase}\;\mathrm{in}\;\mathrm{population}\;\times \;\frac{48}{120}$$
  Total estimated population of Kashmir as on January 2005 calculated as per arithmetic progression formulae was 5762728 with 3028516 males and 2734212 females. This population includes the total number of cases residing in six districts of Kashmir namely Srinagar, Anantnag, Baramulla, Pulwama, Kupwara and Budgam. This population does not include the people residing in Kargil and Leh and it also excludes the migrant population from outside the Kashmir valley. The population estimate thus would be the best possible estimate of the population at the time of the study and not an accurate census which would be beyond the scope of the study.

## RESULTS

A total of 462 incident cases of lung cancer (ICD10: C33, C34) were registered during the study period, with 397 males and 65 females, a M:F ratio of 6.1:1. Thus the annual crude incidence rate of lung cancer was 4.005 per 100 000 population, being 6.55/100 000 in males and 1.18/100 000 in females [Table 1]. The age adjusted incidence rate (adjusted to the world population) was 11.2, being 17.05 in males and 3.21 in females. The truncated age adjusted incidence rate [Table 1] was found to be the highest in the age group of 70-79 years in males and the 60-69 year group in females. More than three-quarter of the cases were observed in the age range from 40 to 69 years. District wise distribution demonstrated the highest incidence in the capital district of Srinagar with the least incidence in Kupwara [Table 2]. The ages of the patients having lung cancer ranged from 14 to 85 years, being 58.28±11.720 in males and 53.26 ±11.763 years in females.

Three hundred and thirty-four (72.3%) cases were current smokers at the time of diagnosis, whereas 43 (9.3%) had been ex-smokers and 83 (17.9%) were non smokers. Majority of male lung cancer patients were smokers i.e., 82.3% (153/186) whereas more than two thirds of the females (24 of the 35, 68.6%) were non-smokers. Of the smokers one-third of the patients smoked cigarettes (water-pipe), 31% used only filtered cigarettes whereas the rest smoked Hukkah, bidi and Hukkah interchangeably. All of the smoking females smoked Hukkah only. Only two patients of lung cancer were using chewable tobacco and both were males. No specific occupations were found to be associated with the development of lung cancer.

**Table 1:** Age specific, age standardized (world), truncated age adjusted incidence rates of lung cancer per 100 000 persons in Kashmir valley (Jan 2004 - Dec 2005)

Age group	Age specific incidence rates		
	Males N (ASPR)	Females N(ASPR)	Total N(ASPR)
0 to 9	0 (0)	0 (0)	0 (0)
10 to 19	2 (0.13) 0 (0)	2 (0.07)	
20 to 29	0 (0.0)	1 (0.12)	1 (0.05)
30 to 39	7 (0.86)	6 (0.83)	13 (0.85)
40 to 49	61 (9.5)	10 (1.90)	71(6.08)
50 to 59	104 (27.28)	12 (3.89)	116 (16.81)
60 to 69	138 (56.95)	26 (13.16)	164 (37.28)
70 to 79	73 (69.58)	8 (9.94)	81 (43.69)
>80	12 (29.45)	2 (6.34)	14 (19.30)
Crude incidence rate (all ages)	397 (6.55)	65 (1.18)	462 (4.005)
Age standardized rate (world)	17.05	3.21	11.21
Truncated age specific rates (40-69 years)	23.95	4.65	15.28

N= Number of patients, ASPR = Age specific incidence rate

**Table 2:** Distribution of district wise crude and age adjusted incidence rate of lung cancer per 100 000 population (Jan 2004 - Dec 2005)

District	Population	Number	CIR	Males	Females	Total
Anantnag	1232681	61	2.47	5.98	1.44	3.85
Baramulla	1235431	60	2.43	5.6	1.48	3.80
Budgam	660007	65	4.92	13.14	2.11	8.11
Kupwara	681981	30	2.20	6. 2	0.92	3.87
Pulwama	688175	55	3.99	10.7	1.84	6.27
Srinagar	1264435	191	7.55	19.3	3.59	12.30

CIR= Crude incidence rate

Histologically, squamous cell carcinoma was the most common histological type (*n* = 312, 67.5%) of the patients and small cell carcinoma constituted 20.8% (*n* = 96) of the cases. There were 14 (3%) cases of adenocarcinoma. Other histological subtypes encountered included large cell type (*n* = 5), carcinoid tumors (*n* = 8), poorly differentiated epithelial tumor (*n* = 4), bronchoalveolar carcinoma (*n* = 1), malignant chondroid tumor (*n* = 4) and undifferentiated tumor (*n* = 18).

## DISCUSSION

Population surveys are superior epidemiological tools, providing true incidence and prevalence of lung cancer in free living population as compared to other studies utilizing mortality rates, autopsy material and hospital admission rates. Although the advantage of the present study is that the sample used for the assessment of the data is assumed to be the closest approximation of a population registry that being multi-institutional and involving all the laboratories and the oncological services of the valley against the backdrop of a practice of all referrals of lung cancer cases to pulmonary or oncology services for management, the limitation about the absoluteness of the data still remains. Several earlier studies have used selected population groups for studying the epidemiology of lung cancer e.g., cohort study of British Physicians,[7] US veterans of Armed Forces,[8] review of 921 consecutive autopsies by Banker in 1957 In India,[1] and analysis of records of 15 teaching institutions in India by Viswanathan *et al* in 1962.[2] However, these studies do not give an idea of the actual disease load in the community and the sample groups used in these studies do not have the same age/sex structure as the general population.

Population-based surveys have been widely used worldwide especially in the developed countries like USA, European Union and Japan.[9–14] In the developing countries, China had started a population-based cancer registry in 1970. In India most of the data in epidemiology of lung cancer has been obtained from hospital data, mostly related to inpatient admissions.[2,5,15–19] Lately population-based cancer registries (PBCR’s) have been set up at various states in collaboration with ICMR (first All India Report, Development of an Atlas of Cancer in India, ICMR[4]. In Kashmir valley an earlier hospital-based study of 35 male lung cancer patients was reported in 1973 by Nafae *et al*.[6] There are no population-based cancer registries and the current data being a multi-institutional data a close approximation of a population-based data. The data based upon hospital surveys are influenced by changes and variations in medical practice, access to care and diagnostic practices. These factors would be especially relevant in India because of the inadequacy of health care and the disproportionate distribution of health care facilities.

In the current study, 462 incident cases of lung cancers were diagnosed histopathologically during the study period, with an overall crude incidence rate of 4.01 per 100 000 persons (males 6.55 and females 1.19). The age standardized (standardized to world population age structure) incidence rates for lung cancer for 100 000 per annum were 11.21 (17.05 per 100 000 for males and 3.21 for females). The truncated age adjusted (standardized to world population, 40-69 years) incidence rates (TR) for lung cancer per 100 000 per annum was 15.28 (23.95 for males and 4.65 for females). These figures suggest that Kashmir valley as a whole is a low risk zone for lung cancer in contiguity with Latin America, Africa, other Asian countries and developing regions of the world. Although the rates are much lower than that of the developed regions of world, the rates are almost similar to the ASR of regions like Costa Rica (12.7),[11,17,20] Setif, Algeria[20] (11.7) and Martinique (11.0) in France.[20] Although the age adjusted incidence rates for males are lower than that of Moari population of New Zealand, USA, Canada and Europe (>50 per 100 000 population), but are higher than age adjusted rates found in region like Mali in Bamaleo (Indonesia), Gamsia, Euador, Algeria, Costa Rica, Peru, Iceland, Norway and Sweden (<15 cases per 100 000 population.[11,20–23]

ASR for lung cancer in females was very low in our present study, when compared with the rest of the world; regions like North West territory of Canada, Hawaii in USA, Scotland in UK; Cuba and Tianjin province in China[22,24,25] have very high ASRs in comparison to our population (17.3 - 72 per 100 000 population)

### Indian comparison

**Figure 1:**
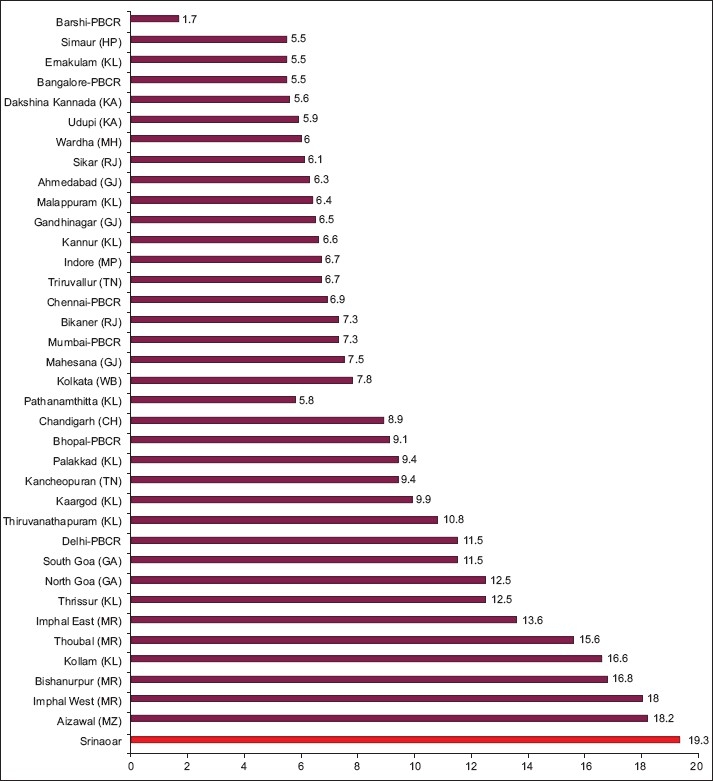
Age standardized incidence rates of lung cancer in India, Srinagar versus other distrcits (Males)

**Figure 2:**
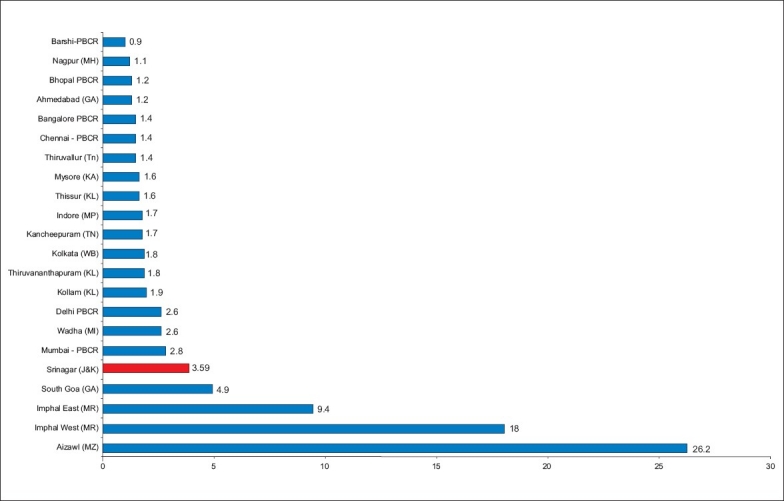
Age standardized incidence rates of lung cancer in India, Srinagar versus other districts (Females)

India come under low risk zone for lung cancer (ASR of (<15/100 000 population). In our study we found that ASR for males and females in Kashmir valley is 11.21/100,000 population, being 17.21/100 000 in males. ASR of lung cancer in Kashmir valley is more than that of regions like Sirmaur (HP) (5.5/100 000), Bangalore (5.5/100 000) and Ernakulam in Kerala (5.5/100 000).[3,4] The areas of the highest ASR in India i.e. the North Eastern region like Aizwal (18.2/100000), Mamit (25.2/100 000) and Imphal west (18.0/100 000) have similar ASR for males as in Kashmir valley.[3,4] If a comparison of districts is made, Srinagar district of the Kashmir Valley has the highest ASR of 19.34/100 000 in males [Figure 1]. Figure 2 depicts the comparison in females. The data comparisons have been made on the assumption that the incidence rates obtained by our study would be closely mimicking those of a population-based registry.

When we compare ASR for females in Kashmir valley as per our study (3.21/100 000) with the rest of India, it is seen that ASR (lung cancer) for females in Kashmir is comparable with the ASR of Mumbai (2.8/100 000), Wardha (2.6/100 000) and Delhi (2.6/100 000).[3,4] It is higher as compared to the ASR (for lung cancer in females) of Nagpur (1.1/100 000), Bhopal (1.2/100 000), Ahmedabad (1.2/100 000), Bangalore and Chennai with ASR of (1.4/100 000) each.[3,4] But ASR (for lung cancer in females) of northeast region of India[3,4] is high as compared to Kashmir valley (9.4 - 26.2/100 000). Besides our study, there were no comparable data available from Kashmir valley. As per our present study, ASR for whole Kashmir valley is 6.5 and when we take into consideration the individual districts, Srinagar district has an ASR of 19.34/100 000, which is the highest ASR compared with any other district in the country. The reasons for this disproportionately high incidence irrespective of high degree of health awareness are being investigated, and higher contribution of environmental tobacco smoking can be postulated to be the causative.

Majority of the cases in the current study were found in sixth and seventh decade. The mean age was 58.28 years for men and 53.26 years for women, the age specific incidence rates being 37.3/100 000 (males 56.95, females 13.2) for the age group 60-69 years and 43.7/100 000 (males 69.6, females 9.94). Truncated age specific rates for 40-69 age group was 15.28/100 000 (males 23.95 and females 4.65). In most of the reported studies from India, the average age for lung cancer is approximately 55 years.[17] In the western countries, the average age falls in the seventh decade of life.[8] The world can in fact be divided into high incidence countries and low incidence countries based on the rate of occurrence of lung cancer, where USA, Canada, new Zealand (Maori population) and Europe come under high incidence countries (>50 cases/10^5^ population), whereas countries like Latin America, Asian countries, Iceland, Norway, Sweden, India, Israel, China can be called as low incidence countries(< 35 cases/10^5^ population).[20,24,26] The sex ratio in our study was 6:1, a male preponderance in conformity with many earlier studies.[2–4,8,10–14,17,18]

In our study, 334 patients (72.2%) were current smokers, 42 (19%) were ex-smokers (left smoking for >10 years), two patients were using chewable tobacco and 83 (17.9%) patients were non smokers. Smoking has been causally linked to lung cancer in various case control and cohort studies reported from 1950 onwards.[7,10,25–27] In our study, we did not quantitatively estimate the lung cancer risk associated with the number of cigarette smoked, the duration of smoking and age. However, Doll and Peto have proposed a quantitative model for lung cancer risk based on the data from the cohort study of British physicians[7,25] which predicted a stronger effect of duration of smoking than the amount smoked per day. Thus, a tripling of the number of cigarettes smoked per day was estimated to triple the risk, while tripling of the duration of smoking was estimated to increase the lung cancer risk hundred fold. Most of the female patient populations in our study were non-smokers (68.5%) while 17.1 and 14.3% were smokers and ex-smokers, respectively. Data for smoking patterns and behavior of females in developing countries continues to be sparse.[11] The data could suggest the role of other etiological factors for the development of cancer in this group of patients and lines of enquiry need to focus upon any environmental exposures, passive smoking and domestic smoke exposures. These factors are being studied as part of another in depth epidemiological study currently under way.

In our study, approximately one-third (30%) of smokers having lung cancer smoked Hukkah (water pipe), 31.7% smoked only filtered cigarette and 8.1% of cancer patients smoked Hukkah, bidi and cigarettes interchangeably. Most of the female patients (5 out of 6 smokers) smoked Hukkah (water pipe). Only five studies in India and three in Pakistan have studied the role of Hukkah smoking as a risk factor for lung cancer. Most of the work published on water pipe comes from Middle East, Syria and Egypt.[28] Unique tobacco habits and risks of lung cancer have attributed Hukkah smoking as the cause of lung cancer in India.[29]

### Histology of lung cancer

Out of 462 cases of lung cancers, 312 (76.5%) cases were of squamous cell variety and 20.77 (*n* = 96 cases) were of small cell variety. Only 14 (3.03%) cases of adenocarcinoma were seen. The comparative histological patterns reported from other studies from India are depicted in Table 3.

In their series of 1009 cases of lung cancers reported from PGIMER, Jindal and Behera reported that 34.3% of the patients had squamous cell carcinoma, 27.8% had anaplastic (small cell carcinoma) whereas 25.9% had adenocarcinoma and rest of the 12.2% were of unclassified variety.[17] Analysis of various studies on lung cancers reported from 1960 onwards in India reveals that squamous histology variety is the most common (averaged mean of 42.6%)[3] followed by small cell carcinoma (averaged mean 25.4%) and adenocarcinoma (averaged mean 18.40%).[6,15,17,30–48] The initial decades of smoking caused epidemic of lung cancer worldwide, squamous cell carcinoma was the most frequent lung cancer and small cell carcinoma was the next most frequent.[20] In the late seventies, the first evidence of shift toward a predominance of adenocarcinoma was noted.[49,50] Hypothesis concerning the shift in histopathology have focused on the potential role of changes in the characteristics of cigarettes and the consequent changes in the doses of carcinogens inhaled.[50] Increased puff volume and subsequent deposition of carcinogens to peripheral air way and alveoli, increased nitrate levels in tobacco smoke and more complete combustion of tobacco forming more of NNK which preferentially Induces adenocarcinoma, are the other postulates.[51] Since the beginning of the 1970s, the new techniques of diagnosis of lung cancer such as fiberoptic bronchoscopy, FNAC, improved stains for detecting mucin hallmark of adenocarcinoma have been introduced, but Thun *et al*, have concluded that “the increase in lung adenocarcinoma since 1960 is more consistent with changes in smoking behaviour and cigarette design than the diagnostic advances”.[52] The predominance of squamous cell carcinoma in our study may be attributed to the smoking behaviour of Kashmiri population where Hukkah smoking is very common form of smoking tobacco both in rural and urbal areas of Population; and any other as yet undefined risk factors.

**Table 3:** Comparative clinical features and cell type patterns as reported in different Indian studies

Authors	Total	M:F	Age	Sm/NS	Squamous cell	Small cell	Adeno Ca	Unclassified
Viswanathan *et al* 1962	95	-	-	-	50.5	-	28.4	21.1
Basu *et al* 1971	24	7	48.3	5	62.5	8.3	25	4.2
Karai *et al* 1967	100	2.4:1	52.1	-	41	-	20	39
Shankar *et al* 1967	20	All M	54	5.7:1	73.3	6.7	20	-
Nagrath *et al* 1970	35	4	47.7	1.9	25.7	-	34.3	40
Reddy *et al* 1970	46	6.4	50	-	50	25	25	-
Guleria *et al* 1971	120	7.6:1	57.2	2:1	46.2	36.5	17.3	-
Jha *et al* 1972	25	2.9	46.6	5.3	44	20	20	20
Garg *et al* 1973	82	-	-	-	46.3	28	20.7	-
Nafae *et al* 1973	25	All M	51	7.3:1	56	20	12	12
Notani *et al* 1974	520	-	-	3.9:1	27.5	11.3	7.3	53.4
Malik *et al* 1976	136	5.2:1	48.5	3.5:1	40.4	21.3	16.9	7.3
Narang *et al* 1977	58	8.7	51.3	4.8	37.9	51.8	10.4	-
Jindal *et al* 1979	150	5.5:1	51.7	2.4:1	32.5	19.3	15.8	21.9
Malhotra *et al* 1986	70	7.8	49.6	4.8	50	17	14.3	17.1
Jindal & Behera 1990	1009	4.5:1	54.3	2.7:1	34.3	27.6	25.9	12.2
Arora *et al* 1990	100	4.05:1	56.4	1:2	27	1	21	41
Rajasekaran *et al* 1993	232	7.9:1	53	2.7:1	72	4.3	3.9	15.1
Gupta *et al* 1998	279	7.41:1	56.7	4.5:1	42.3	32.2	19.9	5.6	
Thippanna *et al* 1998	160	8.4:1	56.4	4:1	67.5	8.8	18.7	5.1
Gupta D *et al* 2001	265	7.8:1	57.5	3.6:1	60	21.5	16.2	2.3
Kashyap *et al* 2001	638	6.17:1	54.6	2.4:1	58.3	-	10.81	-
Present study	462	6.1:1	57.6	4.6:1	67.5	20.8	3.03	4.7

Sm/NS denotes Smoker: Non smoker

Our study is the first systematic study to ascertain the incidence of lung cancer in Kashmir and we conclude that Srinagar, the summer capital of Jammu and Kashmir, has the highest incidence of lung cancer among the males in India. The incidence rates and the comparisons in the present study are based on the assumption of these being the closest approximation of a population-based registry even as concerns about the absoluteness of the data in comparison to a population registry remain the limitation of the study. However, the circumstances of oncological practice in our part of the country make us believe that the data obtained from a population-based study would be very similar. Our study is hoped to be an impetus to various studies addressing the etiological lines of enquiry regarding the high incidence reported as also to the development of strategic screening programs for the early detection of such cases for effective intervention at earlier stages of the disease.
